# Treatment outcomes of gestational choriocarcinoma before and after EMA/CO introduction in Mongolia: a 15-year retrospective cohort study

**DOI:** 10.3389/fonc.2026.1863040

**Published:** 2026-07-17

**Authors:** Ulziijargal Sukhbat, Gandolgor Batsuuri, Eiko Yamamoto, Terkhen Turbat, Oyujin Ulziibaatar, Baatarkhuu Oidov, Ita Daryanti Saragih, Namuun Ganzul, Yen-Liang Liu, Jargalsaikhan Badarch

**Affiliations:** 1 Graduate Institute of Biomedical Sciences, College of Medicine, China Medical University, Taichung, Taiwan; 2 Department of Obstetrics and Gynecology, Mungun guur, Hospital, Ulaanbaatar, Mongolia; 3 Department of Healthcare Administration, Nagoya University Graduate School of Medicine, Nagoya, Japan; 4 Department of Obstetrics and Gynecology, Mongolian National University of Medical Sciences, Ulaanbaatar, Mongolia; 5 Department of Infectious Diseases, Mongolian National University of Medical Sciences, Ulaanbaatar, Mongolia; 6 College of Information Sciences and Technology, Pennsylvania State University, University Park, Pennsylvania, PA, United States; 7 Deparment of Gynecologic Oncology, National Cancer Center, Ulaanbaatar, Mongolia; 8 Master Program for Biomedical Engineering, College of Biomedical Engineering, China Medical University, Taichung, Taiwan; 9 Cancer Biology and Precision Therapeutics Center, China Medical University, Taichung, Taiwan; 10 Mongolia-Japan Hospital, Mongolian National University of Medical Sciences, Ulaanbaatar, Mongolia

**Keywords:** brain metastasis, chemotherapy, EMA/CO, FIGO staging, gestational choriocarcinoma, metastasis

## Abstract

**Objective:**

To evaluate the clinical characteristics, metastatic patterns, and treatment outcomes of gestational choriocarcinoma in Mongolia, with a particular focus on the impact of etoposide–methotrexate–actinomycin D/cyclophosphamide–vincristine (EMA/CO) chemotherapy implementation.

**Methods:**

A retrospective cohort study was conducted including 66 patients diagnosed with gestational choriocarcinoma between 2011 and 2025. Patients were stratified based on FIGO stage and metastatic status. Clinical features, antecedent pregnancy, and treatment outcomes were analyzed. Comparisons between pre-implementation (2011–2017) and post-implementation (2018–2025) cohorts were performed using independent t-test, chi-square test, or Fisher’s exact test as appropriate.

**Results:**

Of the 66 patients, 42.4% (n = 28) had non-metastatic disease (FIGO stage I), while 57.6% (n = 38) presented with metastatic disease (FIGO stages II–IV). Vaginal bleeding was the most common presenting symptom (84.8%). The lungs were the most frequent site of metastasis. Following implementation of the EMA/CO chemotherapy regimen in 2018, higher complete remission rates and lower observed mortality were observed in the post-implementation cohort. Complete remission increased from 69.7% (23/33) to 97.0% (32/33) (p = 0.006), while disease-related mortality decreased from 30.3% (10/33) to 3.0% (1/33) (p = 0.006). Chemoresistance and relapse rates also decreased, although these differences were not statistically significant. Additional interventions, including surgery for residual disease and radiotherapy for brain or lung metastases, were applied when indicated. Despite overall improvements, metastatic disease remained a key determinant of prognosis.

**Conclusion:**

Patients treated after 2018 demonstrated higher complete remission rates and lower observed mortality compared with those treated before 2018. However, due to the retrospective design, absence of adjusted outcome analysis, and baseline differences between treatment-era cohorts, these findings should be interpreted cautiously. Early diagnosis, routine serum β-hCG surveillance, timely referral, and standardized management remain essential for optimizing outcomes in gestational choriocarcinoma.

## 1 Introduction

Gestational Trophoblastic Disease (GTD) encompasses a spectrum of pregnancy-related tumors, including hydatidiform mole, invasive mole, choriocarcinoma, and other trophoblastic tumors (1) (2). These conditions arise from abnormal trophoblastic tissue and are often characterized by excessive serum β-human chorionic gonadotropin (β-hCG), which serves as a vital biomarker for diagnosis and disease monitoring (3, 4). GTD is typically identified through routine pregnancy evaluations, including blood tests and ultrasound imaging, with treatment strategies ranging from evacuation of the molar pregnancy to chemotherapy for malignant cases such as invasive mole and choriocarcinoma (5, 6). Choriocarcinoma, a malignant subtype of GTD, is notable for its aggressive behavior and potential for rapid metastasis. While rare in Western countries, its prevalence is significantly higher in parts of Asia, including Japan and China, suggesting the influence of regional risk factors such as nutritional deficiencies, socioeconomic conditions, and genetic predispositions (7, 8).

Despite the rarity of choriocarcinoma, early detection through serum β-hCG monitoring significantly improves clinical outcomes (9). Serial measurement of β-hCG enables early diagnosis, treatment monitoring, and detection of disease persistence or recurrence, highlighting its essential diagnostic and prognostic role in GTD management (9, 10). Globally, the incidence of gestational choriocarcinoma varies widely (5, 11), reflecting differences in genetic susceptibility, healthcare infrastructure, and diagnostic practices (12, 13). Rates are relatively low in North America and Western Europe, at approximately 0.25 cases per 10,000 pregnancies, whereas higher incidences have been reported in China (3.5 per 10,000) and Southeast Asia (2.3 per 10,000) (14, 15). In Mongolia, the incidence is estimated at 1 case per 10,000 pregnancies, exceeding Western rates but remaining lower than those observed in China, and comparable to regional Southeast Asian estimates (16). This elevated incidence underscores the importance of region-specific epidemiological research, early detection strategies, and optimized clinical management to reduce morbidity and improve outcomes for women affected by this aggressive malignancy (17). These variations highlight the influence of genetic, environmental, and healthcare access factors on disease occurrence and outcomes (18–20).

In Mongolia, for instance, the serum hCG test remains the cornerstone of choriocarcinoma diagnosis, particularly in rural areas where advanced diagnostic tools are limited (21, 22). This disparity underscores the need for enhanced healthcare infrastructure to ensure timely detection and treatment. Choriocarcinoma is a highly malignant gestational tumor, but it is highly curable when diagnosed early, with cure rates exceeding 90% in low-risk cases. Delays in diagnosis or inadequate treatment, however, can lead to metastasis, with mortality rates reaching 10–15% even in developed countries. The disease commonly spreads to the lungs, vagina, and brain, producing diverse clinical manifestations such as vaginal bleeding, respiratory distress, and neurological symptoms (23). Accurate staging and risk stratification, guided by comprehensive diagnostic assessments including imaging, histopathology, and serum beta-human chorionic gonadotropin (β-hCG) testing, are essential for tailoring chemotherapy regimens according to WHO and FIGO scoring systems (24, 25).

In Mongolia, epidemiological data on gestational choriocarcinoma remain limited, and the apparent rarity of the disease may not reflect a truly low incidence but rather underdiagnosis and underreporting. This is likely influenced by disparities in healthcare infrastructure, including limited access to early pregnancy monitoring, inconsistent use of serum biomarkers, and delayed referral to specialized oncology centers. Additionally, the absence of comprehensive national registries and standardized follow-up systems further obscures the true disease burden, contributing to late-stage presentation and incomplete epidemiological characterization.

Consistent with these challenges, more than half of patients in the present study (57.6%) presented with metastatic disease (FIGO stages II–IV), indicating substantial delays in detection and potential gaps in healthcare access. Prior to the implementation of standardized multi-agent chemotherapy, treatment outcomes were suboptimal, particularly among patients with metastatic disease. This study therefore provides one of the first comprehensive longitudinal analyses of gestational choriocarcinoma in Mongolia over a 15-year period (2011–2025), with a specific focus on clinical characteristics, metastatic patterns, and treatment outcomes before and after the introduction of EMA/CO chemotherapy in 2018.

The novelty of this study lies in its integration of long-term clinical data with treatment-era comparison, offering real-world evidence on the impact of standardized chemotherapy in a resource-constrained setting. Importantly, this work contributes to society by highlighting systemic gaps in early diagnosis and access to care, thereby supporting the need for improved screening strategies and healthcare policy interventions. For clinicians, the findings provide practical insights into disease presentation patterns, high-risk metastatic sites, and the effectiveness of EMA/CO chemotherapy, which may guide earlier diagnosis, risk stratification, and optimized treatment decision-making in similar low-resource environments.

## 2 Materials and methods

### 2.1 Study design and patient population

This study was approved by the Ethics Committee of the Mongolian National University of Medical Sciences, School of Medicine (IRB No. 2023/D08). Because of the retrospective nature of the study and anonymized use of medical record data, the requirement for informed consent was waived by the institutional ethics committee. This single-center retrospective cohort study was conducted at the National Cancer Center of Mongolia, a tertiary referral institution serving as the primary oncology center in the country. The study included patients diagnosed with gestational choriocarcinoma between January 2011 and December 2025.

All consecutive cases of female reproductive organ malignancies diagnosed between 2011 and 2025 (n = 6,353) were screened. Among these, 66 patients with confirmed gestational choriocarcinoma were identified and included. For analytical purposes, patients were stratified into nonmetastatic (FIGO stage I) and metastatic (FIGO stages II–IV) groups based on the International Federation of Gynecology and Obstetrics (FIGO) staging system.

**Figure 1:**
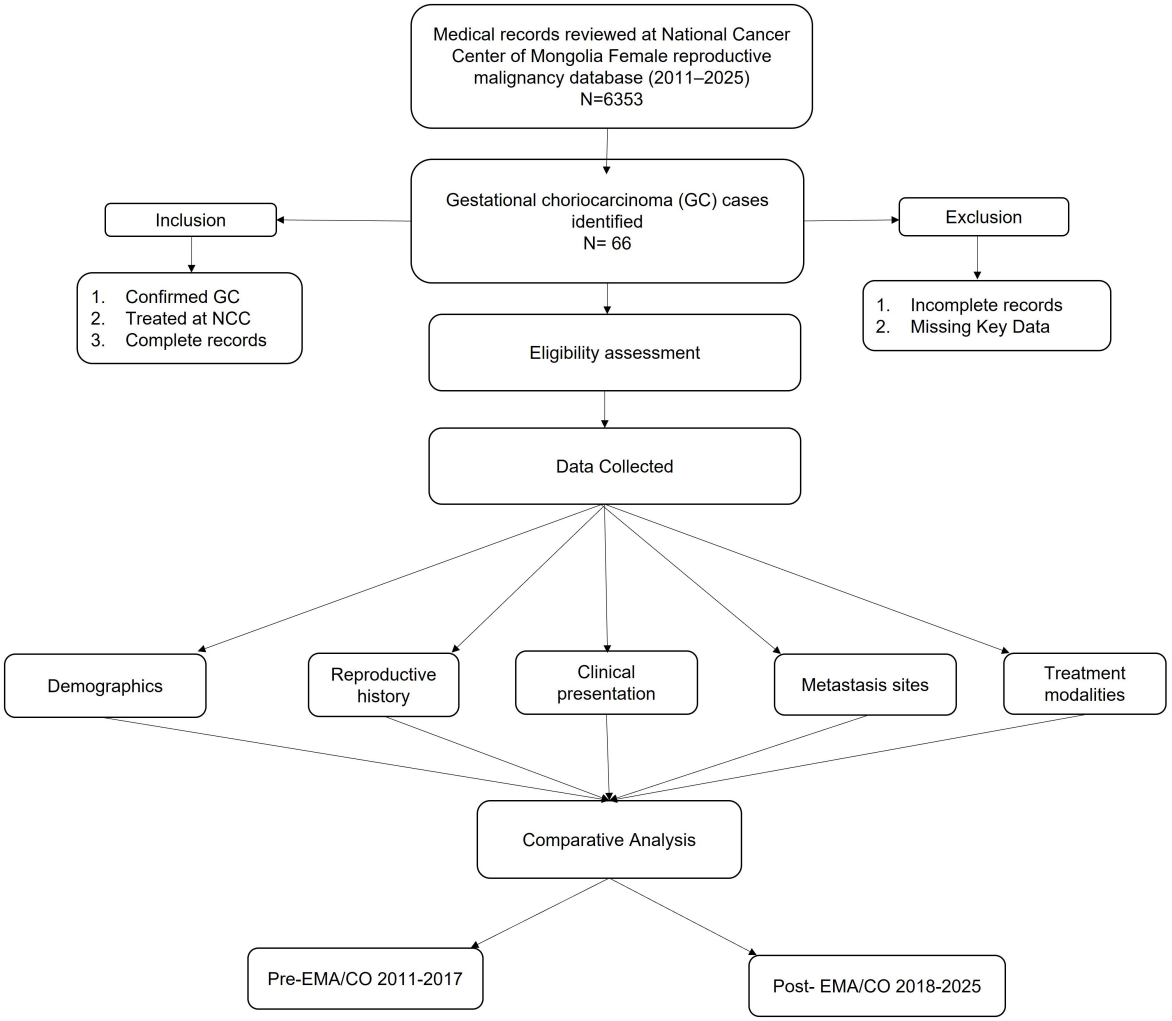
Study design and patient selection flowchart. Medical records from the National Cancer Center of Mongolia were retrospectively reviewed between 2011 and 2025. 66 patients with gestational choriocarcinoma were identified and included for analysis. Patients were stratified according to disease stage, metastatic status, treatment era, and clinical outcomes.

Eligible patients were those with a confirmed diagnosis of gestational choriocarcinoma, supported by histopathological findings and/or established clinical diagnostic criteria, and with complete clinical, treatment, and follow-up records. Diagnosis was established based on histopathological confirmation when available, together with serial serum β-hCG monitoring and clinical evaluation. In patients without tissue confirmation, diagnosis was supported by antecedent pregnancy history, persistently elevated or rising serum β-hCG levels, and imaging findings consistent with gestational trophoblastic neoplasia. Non-gestational choriocarcinoma and other hCG-secreting malignancies were excluded based on clinical history, pathological evaluation, and radiological assessment. Patients were excluded if key clinical data were missing, the diagnosis could not be confirmed, or follow-up information was insufficient to assess treatment outcomes. No additional exclusion criteria were applied (**Figure 1**).

Treatment failure was defined as failure to achieve complete remission despite salvage therapy or death directly attributable to progressive disease during treatment. Clinical variables, including presenting symptoms, treatment modalities, staging, and metastatic patterns, were systematically analyzed. Complete remission was defined as normalization of serum β-human chorionic gonadotropin (β-hCG) levels for at least three consecutive weekly measurements during follow-up.

The EMA/CO chemotherapy regimen consisted of etoposide, methotrexate, and actinomycin D, followed by cyclophosphamide and vincristine, and was administered according to standard clinical protocols.

### 2.2 Data collection and clinical variables

Medical records of patients diagnosed and treated for choriocarcinoma between 2011 and 2025 were reviewed. Variables collected included demographic characteristics, reproductive history, disease characteristics, comorbidities, laboratory parameters, and treatment modalities. Relevant clinical data were systematically extracted from patient medical records using a standardized data collection form and compiled for analysis.

### 2.3 Statistical analysis

Statistical analyses were performed using SPSS version 25.0. Categorical variables were compared using chi-square or Fisher’s exact tests, and continuous variables were analyzed using independent-samples t tests or nonparametric equivalents where appropriate. Multivariable logistic regression analysis was conducted to identify independent predictors of metastasis. Univariable logistic regression analyses were initially performed. Variables with clinical relevance and/or p < 0.10 in univariable analysis were subsequently included in the multivariable model. To reduce the risk of overfitting given the limited sample size, the number of variables included in the model was restricted. Due to the small sample size and limited number of outcome events, multivariable analysis was not performed to evaluate the independent effect of EMA/CO implementation on observed mortality outcomes. Therefore, comparisons between pre- and post-implementation cohorts should be interpreted as exploratory. All statistical tests were two-sided, and a *p* value < 0.05 was considered statistically significant.

## 3 Results

### 3.1 Treatment algorithm and clinical outcomes

**Figure 2:**
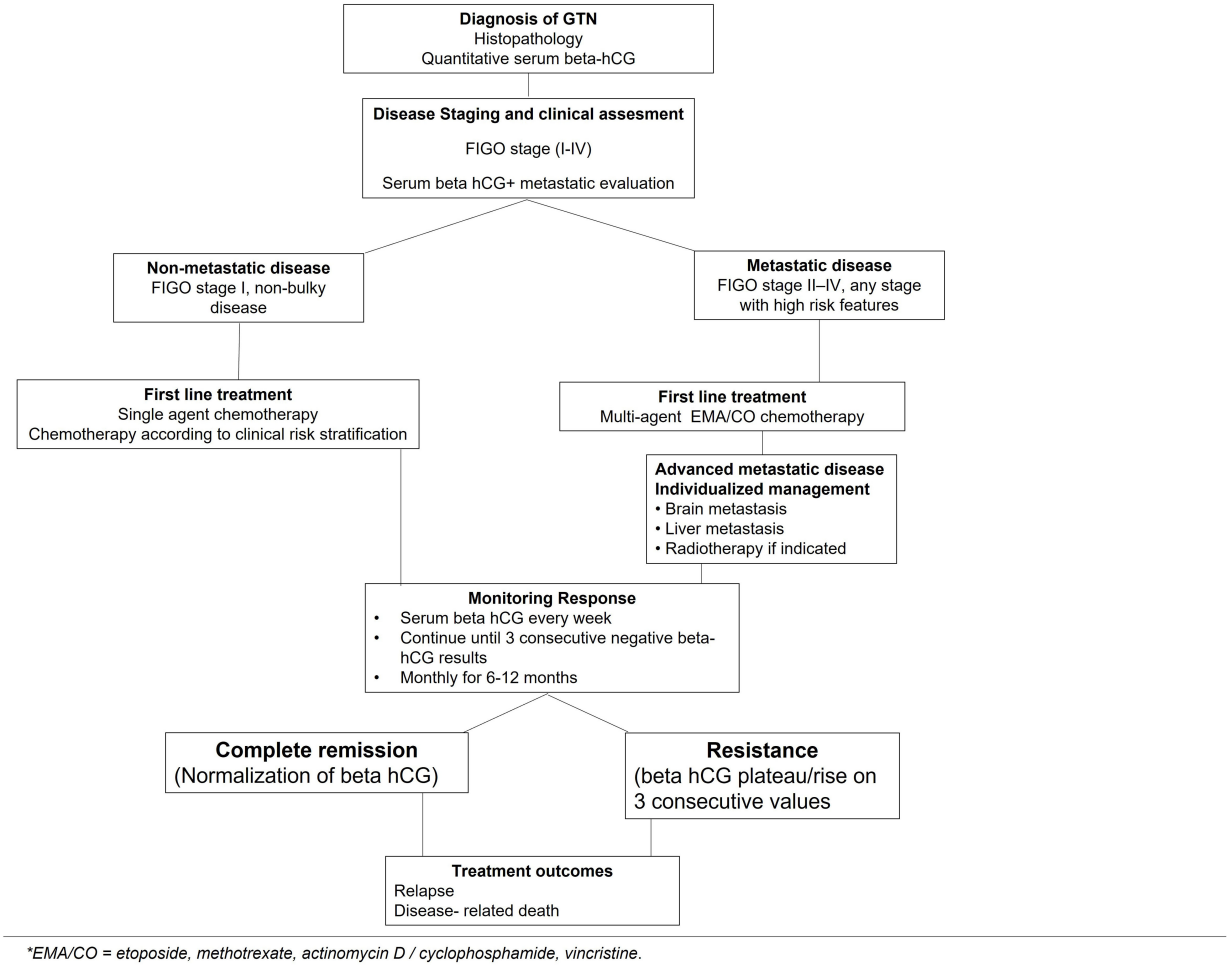
Treatment algorithm for gestational choriocarcinoma with implementation of the EMA/CO regimen.

The treatment algorithm and corresponding clinical outcomes of patients with gestational choriocarcinoma are presented in **Figure 2**. Patients were stratified according to FIGO stage and metastatic status at diagnosis.

Among the 66 patients included in the study, 42.4% (n = 28) had non-metastatic disease (FIGO stage I), while 57.6% (n = 38) presented with metastatic disease (FIGO stages II–IV). Patients with non-metastatic disease were primarily managed with surgical intervention, including uterine evacuation (curettage), and generally demonstrated favorable clinical outcomes.

In contrast, patients with metastatic disease were treated with multi-agent chemotherapy using the EMA/CO regimen. The primary exposure of interest was the implementation of the EMA/CO chemotherapy regimen in 2018. Patients were categorized into pre-implementation (2011–2017) and post-implementation (2018–2025) cohorts. The complete remission rate increased from 69.7% in the 2011–2017 cohort to 97.0% in the 2018–2025 cohort, while mortality decreased from 30.3% to 3.0% (Fisher’s exact test, p = 0.006).

Additional therapeutic interventions, including surgery for residual disease and radiotherapy for brain or pulmonary metastases, were performed when clinically indicated. Despite these improvements, the presence of metastatic disease remained a key determinant of clinical outcome. Overall, these findings suggest that patients treated during the post-implementation period experienced higher remission rates and lower observed mortality, although baseline differences between cohorts may have influenced these outcomes.

### 3.2 Baseline characteristics of patients before and after implementation of EMA/CO chemotherapy

Baseline demographic and clinical characteristics of patients diagnosed before (2011–2017) and after (2018–2025) implementation of the EMA/CO chemotherapy regimen are summarized in **Table 1**. A total of 66 patients were included, with 33 patients in each cohort. The mean age at diagnosis was significantly higher in patients treated after 2018 compared with those treated before 2018 (39.2 ± 8.2 years vs. 32.5 ± 8.3 years, independent t-test, p = 0.002). Disease stage distribution according to the FIGO staging system differed significantly between the two treatment cohorts (Fisher–Freeman–Halton exact test, p < 0.001). In the 2011–2017 cohort, 17 patients (51.5%) presented with Stage I disease, 6 patients (18.2%) with Stage III disease, and 10 patients (30.3%) with Stage IV disease, whereas no Stage II cases were identified. In contrast, among patients treated after 2018, 11 patients (33.3%) were diagnosed with Stage I disease, 10 patients (30.3%) with Stage II disease, 10 patients (30.3%) with Stage III disease, and 2 patients (6.1%) with Stage IV disease. Individual stage comparison demonstrated significant differences in the proportion of Stage II disease (0.0% vs. 30.3%, Fisher’s exact test, p = 0.001) and Stage IV disease (30.3% vs. 6.1%, Fisher’s exact test, p = 0.023), whereas Stage I and Stage III distributions were not significantly different between cohorts. Metastatic disease was observed in 16 patients (48.5%) in the pre-implementation cohort and 22 patients (66.7%) in the post-implementation cohort. Although metastatic disease was more frequent after 2018, this difference did not reach statistical significance (Fisher’s exact test, p = 0.077). These findings demonstrate that the pre- and post-implementation cohorts were not fully comparable at baseline, and these differences should be considered as potential confounding factors when interpreting treatment outcome comparisons.

**Table 1:** Baseline characteristics of patients before and after implementation of EMA/CO chemotherapy.

Variable	2011–2017 (n=33)	2018–2025 (n=33)	P-value
Age, years	32.5 ± 8.3	39.2 ± 8.2	0.002
FIGO stage			<0.001
Stage I	17 (51.5%)	11 (33.3%)	0.213
Stage II	0 (0.0%)	10 (30.3%)	0.0009
Stage III	6 (18.2%)	10 (30.3%)	0.389
Stage IV	10 (30.3%)	2 (6.1%)	0.023
Metastatic disease	16 (48.5%)	22 (66.7%)	0.077
Lung metastasis	12 (36.4%)	11 (33.3%)	0.794
Brain metastasis	4 (12.1%)	0 (0.0%)	0.114
Vaginal metastasis	3 (9.1%)	9 (27.3%)	0.108
Pelvic metastasis	1 (3.0%)	0 (0.0%)	1.000
Kidney metastasis	3 (9.1%)	0 (0.0%)	0.238
Cervical metastasis	0 (0.0%)	1 (3.0%)	1.000
Lymph node metastasis	0 (0.0%)	1 (3.0%)	1.000
Pretreatment β-hCG, median (IQR), mIU/mL	2,633 (639–20,633)	10,000 (814–51,339)	0.672
Pretreatment β-hCG >100,000 mIU/mL	7 (21.2%)	7 (21.2%)	1.000
History of Previous Pregnancies			
Term delivery	27 (81.8%)	28 (84.8%)	1.000
Nulliparous	6 (18.2%)	5 (15.2%)	1.000
Previous adverse obstetric outcomes			
Hydatidiform mole	7 (21.2%)	16 (48.5%)	0.038
Abortion-related pregnancy	14 (42.4%)	5 (15.2%)	0.028
Ectopic pregnancy	0 (0.0%)	1 (3.0%)	1.000
Stillbirth	0 (0.0%)	1 (3.0%)	1.000

*Abbreviations: EMA/CO, etoposide, methotrexate, actinomycin D, cyclophosphamide, and vincristine; FIGO, International Federation of Gynecology and Obstetrics; GTN, gestational trophoblastic neoplasia; hCG, human chorionic gonadotropin.

### 3.3 FIGO stage, metastatic patterns, and stage-specific outcomes

Treatment outcomes before and after implementation of EMA/CO chemotherapy are summarized in **Table 2**. Treatment outcomes were compared between patients treated before implementation of the EMA/CO regimen (2011–2017) and those treated after implementation (2018–2025). The complete remission rate increased significantly from 69.7% (23/33) in the pre-implementation cohort to 97.0% (32/33) in the post-implementation cohort (Fisher’s exact test, p = 0.006). Disease-related mortality decreased from 30.3% (10/33) before 2018 to 3.0% (1/33) after implementation of EMA/CO (Fisher’s exact test, p = 0.006). The proportion of patients demonstrating chemoresistance decreased from 15.2% (5/33) in the pre-implementation cohort to 3.0% (1/33) in the post-implementation cohort; however, this difference did not reach statistical significance (Fisher’s exact test, p = 0.197). Relapse was observed in 2 patients (6.1%) before 2018, whereas no relapse cases were observed after 2018; this difference was not statistically significant (Fisher’s exact test, p = 0.492). Metastatic sites were not mutually exclusive. Several patients presenting with liver or brain metastases also demonstrated concurrent pulmonary metastatic involvement. For staging purposes, patients were categorized according to the highest FIGO stage and most clinically significant metastatic site.

**Table 2:** Comparison of treatment outcomes before and after implementation of EMA/CO chemotherapy.

Outcome	2011–2017 (n = 33)	2018–2025 (n = 33)	Statistical test	P-value
Complete remission (CR)	23 (69.7%)	32 (97.0%)	Fisher’s exact	0.006
Disease-related death	10 (30.3%)	1 (3.0%)	Fisher’s exact	0.006
Chemoresistance	5 (15.2%)	1 (3.0%)	Fisher’s exact	0.197
Relapse	2 (6.1%)	0(0%)	Fisher’s exact	0.492

### 3.4 Prognostic factors and treatment outcomes

Antecedent pregnancy characteristics are summarized in **Table 3**. Hydatidiform mole accounted for 34.7% of cases, while abortion-related pregnancy accounted for 28.9%. According to the WHO/FIGO prognostic scoring system, antecedent pregnancy contributes a score component ranging from 0 to 2, with term pregnancy assigned the highest score component. Complete WHO/FIGO prognostic scores could not be reconstructed because several required clinical variables were unavailable retrospectively.

**Table 3:** Distribution of antecedent pregnancy types and corresponding WHO/FIGO pregnancy score component.

Antecedent Pregnancy	Number (n)	Percentage (%)	FIGO Prognostic Score
Hydatidiform Mole	23	34.7%	0
Abortion (Induced, Spontaneous, Missed)	19	28.9%	1
Term Delivery	3	4.5%	2
Still birth	1	1.5%	2
Ectopic Pregnancy	1	1.5%	1
Non/Unknown	19	28.9%	2
Total	66	100%	

This table represents only the antecedent pregnancy component of the WHO/FIGO prognostic scoring system and does not represent complete WHO/FIGO risk classification.

### 3.5 Independent risk factors for metastasis

Multivariable logistic regression analysis was performed to identify independent risk factors associated with metastasis. As shown in **Table 4**, an initial β-hCG level >100,000 mIU/mL was significantly associated with an increased risk of metastasis (OR = 4.92, 95% CI: 2.2–10.1, p = 0.001). A history of abortion was also identified as a significant risk factor (OR = 2.15, 95% CI: 1.2–4.8, p = 0.028). In contrast, age >40 years was not significantly associated with metastasis (OR = 1.68, 95% CI: 0.8–3.5, p = 0.132). These findings suggest that elevated initial β-hCG levels may be an important predictor of metastatic disease in this cohort, with an approximately five-fold increased odds of metastasis.

**Table 4:** Independent risk factors associated with metastasis in gestational choriocarcinoma.

Variable	Odds ratio (OR)	95% CI	P-value
Initial β-hCG >100,000 mIU/mL	4.92	2.2–10.1	0.001
History of abortion	2.15	1.2–4.8	0.028
Age >40 years	1.68	0.8–3.5	0.132

OR, odds ratio; CI, confidence interval; hCG, human chorionic gonadotropin.

## 4 Discussion

The present study provides important real-world evidence on the epidemiology, clinical characteristics, and treatment outcomes of gestational choriocarcinoma in Mongolia. A key finding is the high proportion of patients presenting with metastatic disease at diagnosis (57.6%), with pulmonary metastasis remaining the most common metastatic site. This pattern may reflect delayed diagnosis, delayed referral, and limited access to specialized care, particularly after the onset of metastatic symptoms. Similar trends have been reported in other resource-limited settings, where lack of structured follow-up and limited access to specialized care contribute to advanced-stage presentation (21, 22). These findings underscore the urgent need for improved early detection strategies, particularly routine surveillance of β-hCG levels following any pregnancy event, including molar pregnancy, miscarriage, or term delivery (6, 11).

Consistent with established FIGO prognostic criteria, the type of antecedent pregnancy was an important determinant of clinical outcome (3, 6). In our cohort, choriocarcinoma following term pregnancy was associated with poorer prognosis compared with post-molar cases (5, 26). This observation aligns with previous studies demonstrating that post-term choriocarcinoma often exhibits more aggressive biological behavior and relative chemoresistance, leading to worse outcomes (27). The underlying mechanisms may involve delayed recognition and differences in tumor biology, emphasizing the importance of vigilant monitoring after all pregnancy outcomes. Complete WHO/FIGO prognostic scoring could not be reconstructed retrospectively because several variables were missing from historical records.

The present study observed higher complete remission rates and lower disease-related mortality in patients treated during the post-2018 treatment period. Although the individual components of the EMA/CO regimen had been available in Mongolia since the early 2000s, standardized institutional implementation of the complete EMA/CO protocol at the National Cancer Center was only consistently adopted beginning in 2018, potentially contributing to improved treatment outcomes observed after this period. However, because measurable baseline differences existed between cohorts, including age and FIGO stage distribution, these findings should be interpreted as an association rather than evidence of the independent therapeutic effect of EMA/CO chemotherapy alone. These findings are consistent with accumulating evidence demonstrating that multi-agent chemotherapy regimens, particularly EMA/CO, are highly effective in treating high-risk gestational trophoblastic neoplasia, including metastatic disease. The marked reduction in mortality observed after 2018 further emphasizes the importance of standardized chemotherapy protocols in improving clinical outcomes.

In contrast, liver metastasis remained a major therapeutic challenge in this study, with an observed mortality rate of 33.3%. Hepatic involvement is widely recognized as an adverse prognostic factor and is associated with aggressive disease, high tumor burden, and increased risk of hemorrhagic complications. Despite the effectiveness of EMA/CO, patients with liver metastases often require a multidisciplinary approach, including interventional radiology techniques such as selective arterial embolization or surgical resection in selected cases. These findings highlight the need for individualized treatment strategies in this high-risk subgroup.

This study represents the first comprehensive cohort analysis of gestational choriocarcinoma in Mongolia using data from the National Cancer Center. Among 6,353 female reproductive organ cancer cases diagnosed between 2011 and 2025, choriocarcinoma accounted for a relatively small proportion, yet its clinical significance remains high due to its rapid progression and metastatic potential. The age distribution in our cohort, with the majority of patients between 30 and 39 years, is consistent with global epidemiological patterns indicating that GTN primarily affects women of reproductive age (28).

Although vaginal bleeding was the most common presenting symptom, a substantial proportion of patients still presented with metastatic disease, with 57.6% classified as FIGO stage II–IV at diagnosis. Similar patterns of late-stage diagnosis have been reported in other developing regions, reflecting limitations in early detection systems and awareness (9, 24).

Metastatic disease was observed in more than half of patients, with the lungs being the most common site of distant spread, followed by the liver and brain. This distribution is consistent with the known hematogenous spread of choriocarcinoma (29). Importantly, our findings suggest that socioeconomic and geographic factors may contribute to delayed diagnosis. Patients residing in rural areas may face greater barriers to timely diagnosis and referral, highlighting potential disparities in healthcare access and the need to strengthen referral systems and healthcare infrastructure in underserved regions (9, 30).

The analysis of mortality trends further emphasizes the clinical importance of this disease. Although choriocarcinoma accounted for a smaller proportion of deaths compared with cervical and ovarian cancers (31), its contribution to mortality remains significant due to its aggressive nature (14, 24). Encouragingly, a declining trend in mortality was observed over time, likely reflecting improvements in diagnostic practices and the implementation of effective chemotherapy regimens.

Serum β-hCG remains a cornerstone in the diagnosis and monitoring of choriocarcinoma. Elevated levels correlate strongly with disease burden and treatment response, making β-hCG an essential biomarker for early detection and follow-up (1, 6). Integrating routine β-hCG monitoring into post-pregnancy care pathways, particularly in rural and resource-limited settings, may facilitate earlier diagnosis and reduce advanced-stage presentation (32, 33).

Detailed treatment-related toxicity data, including Grade 3–4 adverse events, dose reductions, treatment delays, and treatment-related mortality, were not consistently available in retrospective medical records and therefore could not be systematically analyzed. Because toxicity-related laboratory monitoring and formal adverse event grading were not routinely documented during the earlier study period, retrospective classification according to standardized criteria such as CTCAE was not feasible. Future prospective studies incorporating standardized toxicity monitoring and structured adverse event reporting will be necessary to better characterize the safety profile of EMA/CO chemotherapy in Mongolian patients.

Overall, this study highlights the critical importance of early diagnosis, standardized monitoring, and access to modern multi-agent chemotherapy in improving outcomes for patients with gestational choriocarcinoma. Strengthening diagnostic capacity, expanding access to specialized oncology care, and implementing routine β-hCG surveillance programs are essential steps toward reducing disease burden and improving observed mortality in Mongolia.

A major strength of this study is the use of a nationally representative dataset from Mongolia, providing real-world evidence on a rare but clinically significant malignancy in an underreported population. To the best of our knowledge, this is the first retrospective cohort study to systematically investigate gestational choriocarcinoma in Mongolia.

The study highlights important epidemiological and clinical features, including a high proportion of late-stage diagnosis and the potential influence of healthcare access disparities, particularly in rural settings. These findings provide valuable insights into regional disease patterns and underscore the need for improved diagnostic infrastructure and equitable access to care. The high proportion of metastatic cases observed in this study may also be influenced by referral bias, as the National Cancer Center is a tertiary referral institution.

This study has several limitations. First, its retrospective design may introduce selection and information bias. Second, the relatively small sample size from a single national center may limit the generalizability of the findings. In addition, referral bias may have influenced the high proportion of metastatic cases observed, as the National Cancer Center is a tertiary referral institution. Third, some clinical variables and long-term follow-up data were incomplete, which may affect the robustness of outcome assessment. Despite these limitations, this study provides important baseline data on gestational choriocarcinoma in Mongolia and offers valuable insights for future prospective and multicenter studies.

## 5 Conclusion

In conclusion, this study provides important real-world evidence on the clinical characteristics, metastatic patterns, and treatment outcomes of gestational choriocarcinoma in Mongolia. More than half of patients presented with metastatic disease (FIGO stages II–IV) at diagnosis, highlighting the ongoing challenges of delayed detection and limited access to specialized care. In contrast, patients with non-metastatic disease (FIGO stage I) generally demonstrated favorable outcomes. Patients treated after 2018 demonstrated higher complete remission rates and lower observed mortality; however, because measurable baseline differences existed between treatment-era cohorts, these findings should be interpreted as an association rather than evidence of an independent treatment effect.

## Data Availability

The original contributions presented in the study are included in the article/supplementary material. Further inquiries can be directed to the corresponding authors.

## References

[1] Frigo P Lang C Joura E Grabenwoger F Kolbl H . Successful treatment of a high-risk choriocarcinoma in figo stage IV (WHO score 12). Gynecol Obstet Invest. (1997) 44:211–3. doi: 10.1159/000291524 9359651

[2] Vo TM Le KTT Nguyen NH Le CV . Treatment outcomes of methotrexate-resistant post-molar gestational trophoblastic neoplasia: a retrospective cohort study at tu du hospital, Vietnam. Cancer Control. (2026) 33:10732748261427057. doi: 10.1177/10732748261427057 41773064 PMC12957586

[3] Ma N Litkouhi B Mannion CM . Figo stage III metastatic gestational choriocarcinoma developed from an antecedent partial hydatidiform molar pregnancy bearing a numerical chromosomal aberration 68, XX: a case report and literature review. Int J Gynecol Pathol. (2016) 35:162–6. doi: 10.1097/pgp.0000000000000215 26352546

[4] Buckle AE . Methotrexate in treatment of metastasizing chorioncarcinoma: a case report. Br Med J. (1959) 2:1210–3. doi: 10.1136/bmj.2.5161.1210 PMC1991423 13805579

[5] Sato S Yamamoto E Niimi K Ino K Nishino K Suzuki S . The efficacy and toxicity of 4-day chemotherapy with methotrexate, etoposide and actinomycin D in patients with choriocarcinoma and high-risk gestational trophoblastic neoplasia. International Journal of Clinical Oncology. (2020) Jan; 25(1):203–209. doi: 10.1007/s10147-019-01540-9 31520175

[6] Zhao P Lu W . Response: Second curettage versus conventional chemotherapy in avoiding unnecessary chemotherapy and reducing the number of chemotherapy courses for patients with gestational trophoblastic neoplasia: a systematic review and meta-analysis. Int J Gynaecol Obstet. (2024) 164:375–6. doi: 10.1002/ijgo.15235 37924216

[7] Zarate-Correa LC Bejarano-Oliveros MC Reyes-Cardona MJ Vesga-Reyes CE Olaya P Zambrano-Franco JA . Gestational trophoblastic neoplasia with intracardiac metastatic masses. JACC Case Rep. (2026) 31:107845. doi: 10.1016/j.jaccas.2026.107845 41964633 PMC13184880

[8] Xiao L Wan Y Feng W Wang Z . Gestational trophoblastic neoplasia in perimenopausal women: clinical analysis, case series, and literature review. J Vis Exp. (2026) 230. doi: 10.3791/68974 42008430

[9] Francesco P Fabiana C Djaballah Selma A Eleonora L Davide B Silvia S . Choriocarcinoma: diagnosis, treatment and management of a rare germ cell tumour. An update review. Tumori. (2026) 112(3):3008916251408266. doi: 10.1177/03008916251408266 41731693 PMC13250271

[10] Seckl MJ Fisher RA Salerno G Rees H Paradinas FJ Foskett M . Choriocarcinoma and partial hydatidiform moles. Lancet. (2000) 356:36–9. doi: 10.1016/s0140-6736(00)02432-6 10892763

[11] Matsui H Iizuka Y Sekiya S . Incidence of invasive mole and choriocarcinoma following partial hydatidiform mole. Int J Gynecology Obstetrics. (1996) 53:63–4. doi: 10.1016/s0020-7292(96)80014-2 8737309

[12] Mukhoti K Gupta M Ora M Nigam N Yadav T Baskaran R . Isolated pulmonary artery choriocarcinoma masquerading as pulmonary embolism diagnosed by endovascular biopsy: a case report and systematic review. Lung India. (2026) 43:201–6. doi: 10.4103/lungindia.lungindia_19_25 41632559 PMC12995194

[13] Alazzam M Tidy J Osborne R Coleman R Hancock BW Lawrie TA . Chemotherapy for resistant or recurrent gestational trophoblastic neoplasia. Cochrane Database Syst Rev. (2016) 2016:CD008891. doi: 10.1002/14651858.cd008891 26760424 PMC6768657

[14] Crosby C Sanchez-Covarrubias A Ravix J Nair N Sinno A Chery M . Influence of race, ethnicity, and nativity on distribution and outcomes among women with choriocarcinoma in Florida. Cancer Control. (2026) 33:10732748251413803. doi: 10.1177/10732748251413803 41492249 PMC12775359

[15] Yun BS Park EH Ha J Lee JY Lee KH Lee TS . Incidence and survival of gynecologic cancer including cervical, uterine, ovarian, vaginal, vulvar cancer and gestational trophoblastic neoplasia in Korea, 1999-2019: Korea Central Cancer Registry. Obstet Gynecol Sci. (2023) 66:545–61. doi: 10.5468/ogs.23208 37953552 PMC10663396

[16] Golfier F Seckl MJ . From national to international collaboration in gestational trophoblastic disease: hurdles and possibilities. Gynecol Obstet Invest. (2024) 89:254–8. doi: 10.1159/000534321 37827125 PMC11152002

[17] Pietura R Wozniak S Toborek M Kwolek K Pietron N . Three natural pregnancies following embolization of both uterine arteries due to pseudoaneurysms associated with the gestational trophoblastic disease - long-term follow-up. Ginekol Pol. (2024) 95:316–7. doi: 10.5603/gpl.96571 37801617

[18] Smith HO Qualls CR Prairie BA Padilla LA Rayburn WF Key CR . Trends in gestational choriocarcinoma: a 27-year perspective. Obstetrics Gynecology. (2003) 102:978–87. doi: 10.1016/s0029-7844(03)00669-0 14672473

[19] Brinton LA Bracken MB Connelly RR . Choriocarcinoma incidence in the United States. Am J Epidemiol. (1986) 123:1094–100. doi: 10.1093/oxfordjournals.aje.a114337 3706279

[20] Shi YF Li JQ Zheng W Chen XJ Qiao YH Hao M . Survey of gestational trophoblastic disease incidence among 3.6 million pregnancies in China. Zhonghua fu chan ke za zhi. (2005) 40:76–8. 15840282

[21] Yadav DK Ghayour U Acharya B Anjum AS William M Hassan F . Post-term gestational choriocarcinoma presenting with hemorrhagic brain and bilateral renal metastases: a rare case report. Ann Med Surg (Lond). (2025) 87:8845–50. doi: 10.1097/ms9.0000000000003872 41377426 PMC12689088

[22] Ricci C Di Sciascio L Ambrosi F Orsatti A Grillini A Franchini E . New insights into nectin-4 expression in testicular choriocarcinoma and its potential treatment with enfortumab vedotin: analysis of a multi-institutional series and association with clinical-pathological features. Virchows Arch. (2026). doi: 10.1007/s00428-026-04416-2 41559359

[23] Baert T Vermeesch J Timmerman D Vergote I Moerman P . Choriocarcinoma in situ in a partial hydatidiform mole. J Reprod Med. (2016) 61:398–402. 30408390

[24] Alshwayyat S Kamal H Alshwayyat TA Alshwayyat M Hanifa H Alkharabsheh A . Survival rates and predictors in gestational choriocarcinoma: is chemotherapy always the answer? Med (Baltimore). (2025) 104:e46621. doi: 10.1097/md.0000000000046621 41431060 PMC12727261

[25] Braga A Paiva G Ghorani E Freitas F Velarde LGC Kaur B . Predictors for single-agent resistance in figo score 5 or 6 gestational trophoblastic neoplasia: a multicentre, retrospective, cohort study. Lancet Oncol. (2021) 22:1188–98. doi: 10.1016/s1470-2045(21)00262-x 34181884

[26] Benedetto C Borella F Divakar H O'Riordan SL Mazzoli M Hanson M . Response: figo preconception checklist: preconception care for mother and baby. Int J Gynaecol Obstet. (2026) 173:1650–1. doi: 10.1002/ijgo.15446 42132108

[27] Zhou F Kemin L . First-line monodrug chemotherapy in low-risk gestational trophoblastic neoplasia: a network meta-analysis. Front Oncol. (2023) 13:1276771. doi: 10.3389/fonc.2023.1276771 38250546 PMC10796812

[28] Mizushima M Abe M Todo Y Itoh T Narumi M Matsuya M . Hemostasis and life-saving by arterial embolization for a rupture of hepatic metastasis from choriocarcinoma after initiation of EMA/CO therapy: a case report and literature review. Int Cancer Conf J. (2025) 14:446–52. doi: 10.1007/s13691-025-00789-4 41395555 PMC12696255

[29] Alshaikh ABA Al-Kuraishy HM Kafy S Abdelaziz AM Batiha GE . Repurposing statins for choriocarcinoma: targeting the mevalonate pathway to disrupt mechanosignaling and overcome therapeutic resistance. Clin Exp Metastasis. (2026) 43. doi: 10.1007/s10585-026-10395-0 41770291

[30] Bagrecha M Dutta P . Choriocarcinoma presenting with cystic lung metastasis mimicking diffuse cystic lung disease. BMJ Case Rep. (2026) 19. doi: 10.1136/bcr-2025-271406 41741123

[31] Dannehl D Taran FA . Choriocarcinoma: navigating risk, resistance, and remarkable curability. Case Rep Womens Health. (2026) 49:e00772. doi: 10.1016/j.crwh.2025.e00772 41836078 PMC12988509

[32] Illi C Henrich W Hinkson L . Gestational choriocarcinoma figo stage III, score 8 (high-risk) in 38-year-old woman four weeks postpartum. Case Rep Perinat Med. (2025) 14:20240041. doi: 10.1515/crpm-2024-0041 40520651 PMC12165762

[33] Faure-Conter C Rome A Orbach D You B Bolze PA . Gestational trophoblastic diseases and neonatal choriocarcinoma. Bull Cancer. (2026) 113:273–8. doi: 10.1016/j.bulcan.2025.10.007 41407594

