# Correction: Treatment outcomes of gestational choriocarcinoma before and after EMA/CO introduction in Mongolia: a 15-year retrospective cohort study

**DOI:** 10.3389/fonc.2026.1947658

**Published:** 2026-08-05

**Authors:** Ulziijargal Sukhbat, Gandolgor Batsuuri, Eiko Yamamoto, Terkhen Turbat, Oyujin Ulziibaatar, Baatarkhuu Oidov, Ita Daryanti Saragih, Namuun Ganzul, Yen-Liang Liu, Jargalsaikhan Badarch

**Affiliations:** 1 Graduate Institute of Biomedical Sciences, College of Medicine, China Medical University, Taichung, Taiwan; 2 Department of Obstetrics and Gynecology, Mungun Guur Hospital, Ulaanbaatar, Mongolia; 3 Department of Healthcare Administration, Nagoya University Graduate School of Medicine, Nagoya, Japan; 4 Department of Obstetrics and Gynecology, Mongolian National University of Medical Sciences, Ulaanbaatar, Mongolia; 5 Department of Infectious Diseases, Mongolian National University of Medical Sciences, Ulaanbaatar, Mongolia; 6 College of Information Sciences and Technology, Pennsylvania State University, University Park, Pennsylvania, PA, United States; 7 Department of Gynecologic Oncology, National Cancer Center, Ulaanbaatar, Mongolia; 8 Master Program for Biomedical Engineering, College of Biomedical Engineering, China Medical University, Taichung, Taiwan; 9 Cancer Biology and Precision Therapeutics Center, China Medical University, Taichung, Taiwan; 10 Mongolia-Japan Hospital, Mongolian National University of Medical Sciences, Ulaanbaatar, Mongolia

**Keywords:** brain metastasis, chemotherapy, EMA/CO, FIGO staging, gestational choriocarcinoma, metastasis

In the published article, errors were identified in the classification of metastatic disease according to FIGO stage and in several corresponding numerical statements. In addition, two narrative values did not match the values reported in **Tables 1** and **3**. The corrections are as follows.

A correction has been made to the section **Abstract**, *“Results”*.

This sentence previously stated:

“Of the 66 patients, 45.5% (n = 30) had non-metastatic disease (FIGO stage I–II), while 54.5% (n = 36) presented with metastatic disease (FIGO stage III–IV).”

The corrected sentence appears below:

“Of the 66 patients, 42.4% (n = 28) had non-metastatic disease (FIGO stage I), while 57.6% (n = 38) presented with metastatic disease (FIGO stages II–IV).”

A correction has been made to the section **Introduction**, paragraph 5.

This sentence previously stated:

“Consistent with these challenges, more than half of patients in the present study (54.5%) were diagnosed at advanced stages (FIGO III–IV), indicating substantial delays in detection and potential gaps in health care access.”

The corrected sentence appears below:

“Consistent with these challenges, more than half of patients in the present study (57.6%) presented with metastatic disease (FIGO stages II–IV), indicating substantial delays in detection and potential gaps in healthcare access.”

A correction has been made to the section **Materials and methods,** “*2.1 Study design and patient population”*, paragraph 2:

This sentence previously stated:

“For analytical purposes, patients were stratified into non-metastatic (FIGO stage I–II) and metastatic (FIGO stage III–IV) groups based on the International Federation of Gynecology and Obstetrics (FIGO) staging system.”

The corrected sentence appears below:

“For analytical purposes, patients were stratified into non-metastatic (FIGO stage I) and metastatic (FIGO stages II–IV) groups based on the International Federation of Gynecology and Obstetrics (FIGO) staging system.”

A correction has been made to the section **Results**, “*3.1 Treatment algorithm and clinical outcomes*”, paragraph 2:

This sentence previously stated:

“Among the 66 patients included in the study, 45.5% (n = 30) were classified as having non-metastatic disease (FIGO stage I–II), whereas 54.5% (n = 36) presented with metastatic disease (FIGO stage III–IV).”

The corrected sentence appears below:

“Among the 66 patients included in the study, 42.4% (n = 28) had non-metastatic disease (FIGO stage I), while 57.6% (n = 38) presented with metastatic disease (FIGO stages II–IV).”

A correction has been made to the section **Results**, “*3.2 Baseline characteristics of patients before and after implementation of EMA/CO chemotherapy*”:

This sentence previously stated:

“Metastatic disease was observed in 16 patients (48.5%) in the pre-implementation cohort and 24 patients (72.7%) in the post-implementation cohort.”

The corrected sentence appears below:

“Metastatic disease was observed in 16 patients (48.5%) in the pre-implementation cohort and 22 patients (66.7%) in the post-implementation cohort.”

A correction has been made to the section **Results**, “*3.4 Prognostic factors and treatment outcomes*”:

This sentence previously stated:

“Hydatidiform mole and abortion-related pregnancy each accounted for 39.4% of cases.”

The corrected sentence appears below:

“Hydatidiform mole accounted for 34.7% of cases, while abortion-related pregnancy accounted for 28.9%.”

A correction has been made to the section **Discussion**, paragraph 1.

This sentence previously stated: “A key finding is the high proportion of patients presenting with metastatic disease at diagnosis (54.5%), with a predominance of FIGO stage III disease, particularly pulmonary metastasis (47.0%).”

The corrected sentence appears below:

“A key finding is the high proportion of patients presenting with metastatic disease at diagnosis (57.6%), with pulmonary metastasis remaining the most common metastatic site.”

A correction has been made to the section **Discussion**, paragraph 6.

This sentence previously stated:

“Although vaginal bleeding was the most common presenting symptom, a substantial proportion of patients still presented with advanced disease, with 54.5% classified as FIGO stage III–IV at diagnosis.”

The corrected sentence appears below:

“Although vaginal bleeding was the most common presenting symptom, a substantial proportion of patients still presented with metastatic disease, with 57.6% classified as FIGO stage II–IV at diagnosis.”

A correction has been made to the section **Conclusion**.

This sentence previously stated:

“More than half of patients presented with metastatic disease (FIGO stage III–IV) at diagnosis, highlighting the ongoing challenges of delayed detection and limited access to specialized care. In contrast, patients with non-metastatic disease (FIGO stage I–II) generally demonstrated favorable outcomes.”

The corrected sentence appears below:

More than half of patients presented with metastatic disease (FIGO stages II–IV) at diagnosis, highlighting the ongoing challenges of delayed detection and limited access to specialized care. In contrast, patients with non-metastatic disease (FIGO stage I) generally demonstrated favorable outcomes.

In the published article, the classification of non-metastatic and metastatic disease in **Figure 2** was incorrect. The figure incorrectly stated “Non metastatic disease – FIGO Stage I–II” and “Metastatic disease – FIGO Stage III–IV.

**Figure 2:**
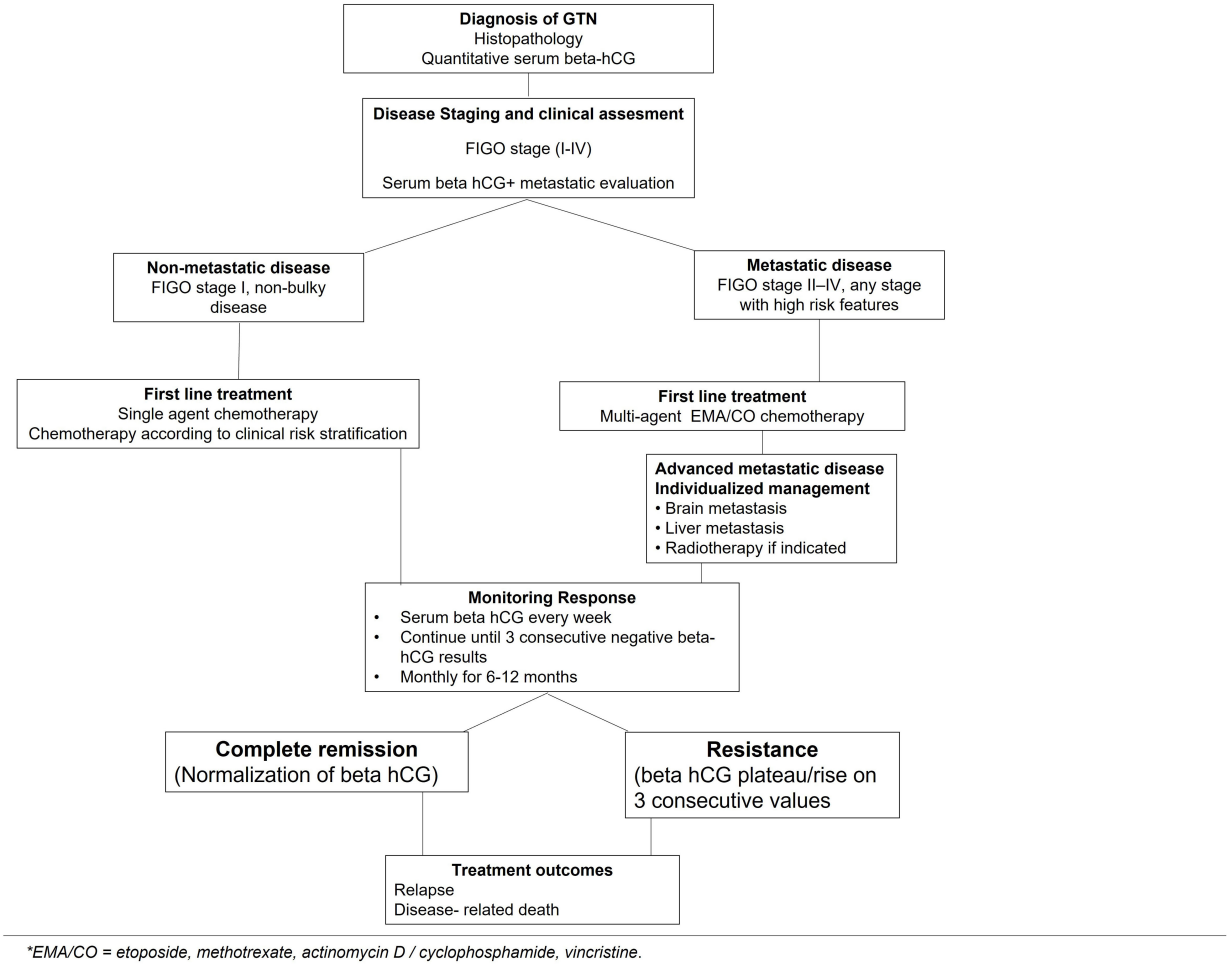
Treatment algorithm for gestational choriocarcinoma with implementation of the EMA/CO regimen.

The correct classification is “Non- metastatic disease – FIGO Stage I” and “Metastatic disease – FIGO Stages II–IV.” **Figure 2** has now been corrected accordingly.

The original version of this article has been updated.

